# Ecological Study of Community-Level Factors Associated With Chronic Mountain Sickness in the Young Male Chinese Immigrant Population in Tibet

**DOI:** 10.2188/jea.JE20110058

**Published:** 2012-03-05

**Authors:** Xiaoxiao Li, Tao Pei, Haotong Xu, Fasheng Tao, Haiyan You, Yan Liu, Yuqi Gao

**Affiliations:** 1 Department of Health Service, College of High Altitude Military Medicine, Third Military Medical University, Chongqing, P. R. China; 2 Department of Health Service, Urumqi General Hospital of Lanzhou Military District, Urumqi, Xinjiang, P. R. China; 3 Key Laboratory of High Altitude Medicine, Ministry of Education, Third Military Medical University, Chongqing, P. R. China; 4 Key Laboratory of High Altitude Medicine, PLA, Chongqing, P. R. China; 5 Department of Radiology, Nanchong Central Hospital of North Sichuan Medical College, Nanchong, Sichuan, P. R. China; 6 Department of Health Service, Ngari Military Subdistrict, Tibet, P. R. China

**Keywords:** chronic mountain sickness, community factor, high altitude, immigrant, Tibet

## Abstract

**Background:**

Chronic mountain sickness (CMS) is a complex medical and public health problem that seriously affects highland immigrants. This study investigated relationships between community-level factors and CMS.

**Methods:**

In this ecological study, data on age- and ethnicity-standardized CMS rates, community factors, and controlling variables were obtained from 2009–2010 surveys of 108 Chinese highland military units. Associations among variables were examined using correlation tests, analyses of covariance, and logistic regression.

**Results:**

The rate of CMS ranged from 1.25% to 36.58% (mean: 14.65%, standard deviation: 8.15%) among military units. Partial correlation tests indicated that medicine expenditure was strongly negatively correlated with CMS (*r* = −0.267, *P* = 0.005). Analyses of covariance indicated that communities with oxygen-generating systems had lower CMS rates (*F* = 9.780, *P* = 0.002), whereas urban location (*F* = 5.442, *P* = 0.022) and construction duty (*F* = 4.735, *P* = 0.011) were associated with higher CMS rates. The multiple logistic model showed that medicine expenditure (OR = 0.897, *P* = 0.022), oxygen-generating system (available vs unavailable: OR = 0.827, *P* = 0.020), community type (urban vs rural: OR = 1.228, *P* = 0.019), and occupation (construction vs logistics: OR = 1.240, *P* = 0.029) were significantly associated with CMS.

**Conclusions:**

We identified community-level, health-related factors that were associated with CMS among young male immigrants. To alleviate the burden of CMS in these highland immigrant populations, further investment should be made in medicine and oxygen-generating systems, and preventive interventions should be implemented among construction workers. Further research should investigate the effects of urbanization on CMS development.

## INTRODUCTION

More than 140 million people worldwide live at altitudes above 2500 m,^1^ and many others travel to highland areas for business and recreation. An increasing number of immigrants have entered Tibet, the world’s largest highland area, to guard and develop the territory. Most immigrants are young Chinese men from low-altitude areas and include workers, miners, military servicemen, and officials. Among natives of lower altitudes, prolonged residence in the highlands is a risk factor for chronic mountain sickness (CMS), also called Monge disease.^2^ CMS is characterized by an elevated hematocrit, as compared with that in individuals residing at sea level, and can progress to cardiac failure or neurological disorders.^3^ CMS has been found in 5% to 18% of populations residing at or above 3200 m on the South American Altiplano and the Tibetan plateau.^4^^–^^6^ CMS rates are much higher among immigrant Han (ethnic Chinese) individuals than among native Tibetans.^7^^,^^8^

CMS is a complex medical and public health problem that is associated with personal suffering and workforce loss and requires public health policies that include sufficient control and prevention measures. However, healthcare services available to highland immigrant communities differ markedly from those at lower altitudes. The natural environment is harsh, the local civil service system is sometimes underdeveloped, and the local culture can be unfamiliar to immigrants. It is difficult to provide advanced healthcare in this context, and even primary care may be inadequate. Due to insufficient resources and transportation difficulties, the supply of medicine is inconsistent, especially in remote or small immigrant communities. It is also difficult to install, maintain, and support sanitation facilities in highland areas. The inadequacy of health-service human resources is also a serious problem. Deployment of skilled medical staff to the highlands for long service periods is more difficult than for other skilled workers, due to the long and costly training and, especially, the personal opportunity cost for such staff. It is thus extremely expensive to support and enhance highland health services. The implementation of reasonable policies and strategies to improve health services for highland immigrant communities is a critical factor in efforts to control CMS.

Some authors have emphasized the importance of analysis at the “whole population” level,^9^ and successful disease control must take community-level factors into account. Many community factors have been associated with health, including medical-resource distribution, economic context, sanitation facilities, transportation infrastructure, and culture.^10^^–^^14^ An evaluation of the effect of immigrant community-level factors on CMS can be used to estimate the return on investment of health-promotion measures and other policies designed to control and prevent CMS. Such research may also provide a powerful means of communicating the value of risk-factor modification to public policy-makers. However, most previous studies have viewed CMS as a biomedical problem and have devoted insufficient attention to community-level factors. We thus performed an ecological study to identify associations between community-level, health-related factors and CMS prevalence using data collected from immigrant communities of young Chinese men in Tibet.

## METHODS

### Ethical considerations

This study complied with the Helsinki Declaration and was approved by the Ethical Review Board of the Third Military Medical University (Approval number: 2009020016). The researchers had no contact with the individuals whose data were accessed, and all data were anonymized before retrieval and analysis.

### Study design and participants

This ecological study used contemporary survey data collected by 2 annual cross-sectional, population-based surveys conducted in October 2009 (Qinghai–Tibet Road) and May–June 2010 (Xinjiang–Tibet Road) by the Chinese Highland Medical Corps and the authors of this paper. Eleven community-level, health-related factors were examined to determine their risk-related or protective effects on CMS rates: (1) residential altitude, (2) number of medical officers and (3) medical soldiers/100 persons, (4) medicine expenditure, (5) hospital transfer time, (6) smoking and (7) drinking rates, (8) use of oxygen-generating and (9) water purification systems, (10) occupation, and (11) community type. Variables 1 through 7 were continuous; 8 through 11 were categorical. Two to 3 coded categories were established for each categorical variable. Because altitude is a determining factor for CMS development,^15^^,^^16^ it was treated as a controlling variable in this study.

To ensure that small to moderate correlations (*r* = 0.25, α = 0.05, 1 − β = 0.80) would be detected, adequate sample size was estimated using the following formula: *n* = 4{(*u*_α_ + *u*_β_)/ln[(1 + *r*)/(1 − *r*)]}^2^ + 3.^17^ To accommodate potentially missing values, we increased the original estimated sample size by 10%. We determined that an adequate sample would thus include 108 units. The requirement for at least 3 × 32 (α = 0.05, 1 − β = 0.80, δ/σ = 0.80) values for analyses of variance, including categorical factors, was met by this sample size. For logistic regression with 24 (ie, 2 × 2 × 2 × 3) detailed outcomes, the samples analyzed for each outcome should include 10 or more CMS cases, and the CMS rate should be at least 5%. A population of 4400 individuals was thus required for this study. Assuming an average population of 50 individuals in each community, 108 communities should contain about 5040 individuals, which was sufficient for analysis. Our final sample contained 854 (14.3%) individuals with CMS and 5111 (85.7%) individuals without CMS.

All units included in sampling had been established on the Tibetan plateau for longer than 5 years, had populations exceeding 30 individuals, and had available data for all study variables. According to the ratio of total unit numbers in the 2 regions, sample quotas were 43 (Qinghai–Tibet Road) and 65 (Xinjiang–Tibet Road). A 2-stage sampling method was used to select units in each study area. First, the study area was divided into groups according to administrative region, and the sample quota was distributed among these groups according to the proportion of unit numbers in each group. Secondly, sample units were selected in each group using a simple random-sampling method. All data were obtained from questionnaire-based surveys or interviews with unit leaders. All surveys and interviews were performed by trained investigators using standard investigating forms. During the survey, all community-level information was strictly reviewed, and information was gathered to eliminate any missing values before the observers left the unit.

### Measurement of variables

CMS was diagnosed using the criterion of excessive erythrocytosis (Hb ≥210 g/L in males)^18^ in individuals who had lived in the highlands for longer than 1 year. Hemoglobin values were measured using the cyanmethemoglobin method. To calculate age- and ethnicity-standardized CMS rates, we first counted CMS cases in each unit separately for each age and ethnicity, then calculated age- and ethnicity-specific CMS rates that were weighted according to the age and ethnicity distributions of each unit, and finally calculated aggregated community rates. Similarly, the numbers of individuals with and without CMS were standardized using age and ethnicity distributions. Calculations of CMS prevalence excluded native highlanders and new immigrants (<1 year highland residence).

The residential altitude (in km) of each immigrant community was extracted from the management records of each unit. Each Chinese highland unit usually has an affiliated health-service organization, termed *weishengsuo* or *weishengshi*, that contains several medical officers and/or medical soldiers. The number of medical officers (equivalent to primary care physicians) and medical soldiers (equivalent to nurses or healthcare workers) per 100 persons was calculated by dividing the number of officers/soldiers in each unit by the total number of unit personnel. Medicine expenditure was computed as Chinese yuan spent annually on medicine per capita in each unit. Data were standardized using the transformation *z* = *x*/σ, where *z* is the standard score, *x* is a raw value to be standardized, and σ is the standard deviation of the population. Because road conditions differ markedly among highland areas, hospital accessibility could not be described simply by calculating the distance from a unit to a hospital. We thus used hospital transfer time (in hours) under normal conditions to describe hospital accessibility. Smoking and drinking rates were calculated by dividing the number of current smokers and drinkers in each unit by the number of total unit personnel. Oxygen-generating systems, which consist of concentrated oxygen-supply devices linked by pipes to masks at each unit member’s bed, were classified as available (installed and providing regular oxygen supply) or unavailable (absent or not functioning due to lack of maintenance or supply). Similarly, water purification systems, which consist of centralized water supply systems employing flocculation, sedimentation, and filtration processes (or equivalents), were also classified as available or unavailable. Each unit’s occupation was classified as guard, construction, or logistics, according to its main task. Community type was classified as urban or rural, according to the local government’s definition.

### Statistical analyses

We plotted trends in CMS prevalence rates with increasing altitude and generated descriptive statistics of independent and dependent variables for the sample population. To investigate associations between CMS prevalence rates and each continuous variable, we computed partial correlation coefficients using residential altitude as a controlling variable to adjust for any differences in the severity of hypobaric hypoxia. Analyses of covariance (ANCOVA) examined differences in CMS prevalence between categories in each binary or nominal variable, using altitude as a covariate.

We also performed separate binary logistic regression analyses using CMS diagnosis outcome (absent = 0, present = 1) as the dependent variable and each community factor as the independent variable. These analyses were weighted by the number of CMS and non-CMS cases and adjusted for altitude. All variables found to be associated significantly (*P* < 0.10) with CMS rates in the adjusted separate models were entered as independent variables into a multiple logistic regression model. Altitude was also entered into this model as a controlling variable. The odds ratios (ORs), 95% confidence intervals (95% CIs), and *P*-values for each variable are reported for separate and multiple regression analyses.

The Kolmogorov–Smirnov (K–S) test was used to examine the normality of each continuous variable. Log transformations were used for non-normal variables. To analyze categorical variables, we transformed them into dummy variables. To assess the goodness-of-fit of multiple regression models, Hosmer–Lemeshow tests were performed. The fulfillment of absence of collinearity in the multiple regression models was tested by calculating variance inflation factors (VIFs). For all statistical analyses, a *P* value less than 0.05 was considered to indicate statistical significance; a *P* value less than 0.10 was considered to indicate an acceptable selection level. Data were analyzed using SPSS software (ver. 13.0; SPSS, Inc., Chicago, IL, USA).

## RESULTS

CMS prevalence increased with altitude in the 3 community types (Figure). Table 1 shows the descriptive statistics for the sample population. The continuous variables varied widely among these communities: age- and ethnicity-standardized CMS prevalence rates ranged from 1.25 to 36.58 per 100 individuals; residential altitudes ranged from 3.175 to 5.380 km; the number of medical officers and medical soldiers per 100 persons ranged from 1.00 to 5.70 and 1.02 to 7.00, respectively; standardized scores for medicine expenditure ranged from 4.96 to 9.20; smoking and drinking rates ranged from 40.48 to 69.72 and 5.03 to 19.99 per 100 persons, respectively; and hospital transfer time ranged from 0.1 to 71.6 hours. All continuous variables were distributed normally, except hospital transfer time (*P* = 0.001). Log transformation brought hospital transfer time within the range of normality (*P* = 0.089).

**Figure.**
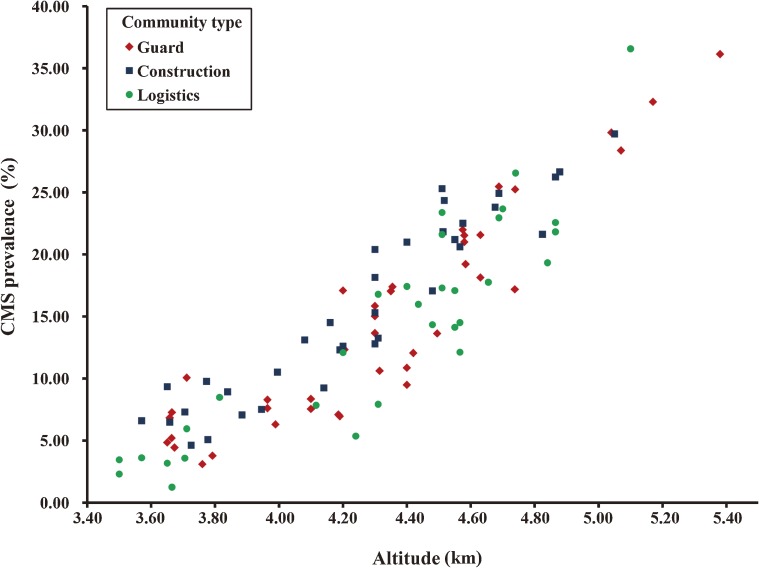
Chronic mountain sickness (CMS) prevalence rates according to altitude in 3 types of communities

**Table 1.** Characteristics of highland immigrant communities (*n* = 108)

Continuous variables	Mean	SD
Chronic mountain sickness rate (%)	14.65	8.15
Altitude (km)	4.27	0.45
Medical officers/100	2.48	0.99
Medical soldiers/100	3.16	1.18
Medicine expenditure^a^	7.47	1.00
Hospital transport time (hours)	17.13	18.89
Smoking rate (%)	56.29	8.55
Drinking rate (%)	12.67	4.48

Categorical variables	*n*	%

Oxygen-generating system		
Unavailable	56	51.85
Available	52	48.15
Water purification system		
Unavailable	62	57.41
Available	46	42.59
Community type		
Rural	64	59.26
Urban	44	40.74
Occupation		
Guard	40	37.04
Construction	35	32.41
Logistics	33	30.56

^a^Standardized score. SD: standard deviation.

Table 2 shows the results of correlation analyses between CMS prevalence rates and each continuous variable and ANCOVA to detect differences between categories in CMS prevalence for each binary or nominal factor. After adjusting for altitude, only higher medicine expenditure (*r* = −0.267, *P* = 0.005) retained a significant inverse correlation. Using altitude as a covariate, ANCOVA showed that CMS prevalence was significantly higher (*P* = 0.022) in urban communities (mean: 15.70/100, 95% CI: 14.60–16.80) than in rural communities (mean: 13.97/100, 95% CI: 13.09–14.85). CMS prevalence was significantly lower (*P* = 0.002) in communities with available oxygen-generating systems (mean: 13.57/100, 95% CI: 12.63–14.52) than in those in which such systems were unavailable (mean: 15.67/100, 95% CI: 14.76–16.58). Construction units had a significantly higher (*P* = 0.011) CMS prevalence (mean: 16.12/100, 95% CI: 14.97–17.26) than did guard (mean: 14.05/100, 95% CI: 12.87–15.23) and logistics (mean: 13.89/100, 95% CI: 12.82–14.96) units. No significant difference was found in the homogeneity of variances in any ANCOVA (*P* > 0.05).

**Table 2.** Correlations between chronic mountain sickness (CMS) prevalence and each continuous variable, and analysis of variance in CMS prevalence between different categories of each categorical variable (*n* = 108)

Continuous variable	Covariate not entered		Covariate entered	
	
	*r*^a^	*P*	*r*^b^	*P*
Altitude (km)	0.903	<0.001		
Medical officers/100	−0.092	0.346	−0.063	0.521
Medical soldiers/100	−0.035	0.717	−0.094	0.337
Medicine expenditure^c^	−0.199	0.039	−0.267	0.005
Hospital transport time^d^	0.359	<0.001	−0.062	0.525
Smoking rate (%)	0.057	0.558	0.070	0.474
Drinking rate (%)	−0.014	0.883	0.049	0.614

Categorical variable	Covariate not entered		Covariate entered	
	
	*F*^e^	*P*	*F*^f^	*P*

Oxygen-generating system	12.755	0.001	9.780	0.002
Water purification system	0.161	0.689	0.320	0.573
Community type	8.607	0.004	5.442	0.022
Occupation	0.607	0.547	4.735	0.011

^a^Pearson’s correlation coefficient between CMS prevalence and each continuous variable (unadjusted); ^b^partial correlation coefficient between CMS prevalence and each continuous variable (adjusted for altitude); ^c^standardized score; ^d^log-transformed; ^e^*F*-value of analysis of variance between categories in each categorical variable; ^f^*F*-value of analysis of covariance between categories in each categorical variable, using altitude as a covariate.

Before adjusting for altitude, separate logistic regression analyses found that the following community factors were associated significantly with CMS (*P* < 0.10): medical officers per 100 persons, medicine expenditure, hospital transport time, availability of oxygen-generating system, community type, and occupation. After adjusting for altitude, medical officers per 100 persons and hospital transport time were no longer significant (*P* > 0.10), while the other factors remained significant (Table 3). We entered all significant variables from the separate models into a multiple logistic regression model and found that these variable remained significant. The 4 factors were moderately but consistently associated with CMS rates in the multiple regression model (Table 4). The Hosmer–Lemeshow test showed that the multiple regression model fit well (*P* = 0.716). The VIF of independent variables ranged from 1.143 to 1.606 (mean: 1.383), indicating an acceptable absence of collinearity.

**Table 3.** Separate logistic regression analyses of the relationship between chronic mountain sickness and immigrant community factors, adjusted for altitude

Variable	Unadjusted		Adjusted^b^	
	
	OR (95% CI)^c^	*P*	OR (95% CI)^c^	*P*
Altitude (km)	4.234 (3.517, 5.098)	<0.001		
Medical officers/100	0.928 (0.864, 0.998)	0.043	0.992 (0.918, 1.071)	0.835
Medical soldiers/100	0.967 (0.909, 1.030)	0.297	0.990 (0.928, 1.056)	0.753
Medicine expenditure^a^	0.647 (0.599, 0.698)	<0.001	0.873 (0.797, 0.956)	0.003
Hospital transport time (10 hours)	1.081 (1.041, 1.122)	<0.001	0.972 (0.930, 1.015)	0.194
Smoking rate (%)	1.004 (0.995, 1.013)	0.387	1.003 (0.994, 1.011)	0.534
Drinking rate (%)	0.996 (0.980, 1.013)	0.641	1.003 (0.987, 1.019)	0.737
Oxygen-generating system				
Unavailable (ref.)	1		1	
Available	0.647 (0.558, 0.750)	<0.001	0.804 (0.690, 0.937)	0.005
Water purification system				
Unavailable (ref.)	1		1	
Available	1.068 (0.923, 1.236)	0.378	1.035 (0.891, 1.202)	0.651
Community type				
Rural (ref.)	1		1	
Urban	0.734 (0.631, 0.854)	<0.001	0.823 (0.696, 0.973)	0.023
Occupation				
Logistics (ref.)	1		1	
Guard	1.082 (0.904, 1.294)	0.390	1.042 (0.868, 1.252)	0.657
Construction	1.198 (0.996, 1.439)	0.055	1.290 (1.068, 1.559)	0.008

OR, odds ratio; CI, confidence interval. ^a^Standardized score; ^b^separate logistic regression models adjusted for altitude; ^c^standardized odds ratios and 95% confidence intervals for continuous variables.

**Table 4.** Multiple logistic regression analysis of the relationship between chronic mountain sickness and significant immigrant community factors (model including all variables)

Variable	OR	95% CI	*P*
Residential altitude (km)^a^	3.970	(3.107, 5.074)	<0.001
Medicine expenditure^b^	0.897	(0.817, 0.985)	0.022
Oxygen-generating system			
Unavailable (ref.)	1		
Available	0.827	(0.704, 0.970)	0.020
Community type			
Rural (ref.)	1		
Urban	1.228	(1.034, 1.460)	0.019
Occupation			
Logistics (ref.)	1		
Guard	1.104	(0.910, 1.340)	0.316
Construction	1.240	(1.022, 1.504)	0.029

OR, odds ratio; CI, confidence interval. ^a^Control factor; ^b^standardized odds score.

## DISCUSSION

To our knowledge, this study is the first to analyze the relationship between community-level, health-related factors and CMS prevalence rates in the highland immigrant population of Tibet. The 108 immigrant communities included in this study are considered to be representative of the young male Chinese immigrant population. Data were collected by trained investigators using rigorous criteria and extracted from military medical records, which are likely to have high validity.

Several social factors have been found to be closely related to chronic disease.^19^^,^^20^ The present study identified community-level, health-related factors that were significantly associated with the development of CMS in immigrants. CMS rates were significantly lower in immigrant communities with greater medicine expenditures and oxygen-generating system availability. In contrast, construction duty was associated with a higher CMS rate as compared with guard and logistics duty at the population level. Urban location was also associated with significantly increased CMS rates in immigrant communities. We offer our interpretation of these findings below.

Firstly, medicine supplies (as measured by expenditure) played an important role in decreasing CMS risk in highland immigrant communities. Several Western and traditional Chinese medicines have been shown to effectively treat CMS^21^^–^^25^; thus, additional preventive and therapeutic medicine for CMS should be provided to immigrant populations. Medical expenditure per capita ranged widely among the units in our study (standardized scores: 4.96–9.20) and was clearly unequal among immigrant communities. Immigrant communities with lower medicine expenditure are more likely to be unable to fulfill the medical needs of residents, which places them at a disadvantage in efforts to control CMS development. Efforts should thus be made to ensure that all communities have sufficient medicine resources to provide at least the present average expenditure (standardized score: 7.47) for their members.

Secondly, oxygen supply also plays an important role in CMS prevention. Chronic hypoxic stress can affect bone marrow, causing erythrocytosis.^4^^,^^26^ Although an increase in hemoglobin facilitates oxygen transport at high altitudes, excessive erythrocytosis may cause CMS.^27^ Oxygen-generating systems provide high concentrations of oxygen to alleviate hypoxic stress.^18^ Although such systems cannot provide oxygen all day, they can somewhat improve individual health. Unfortunately, although some military units had completed installation of oxygen-supply systems, they lacked the maintenance or electrical supply to ensure that these systems were functioning properly.

Construction workers were more likely than others to develop CMS. Construction labor is associated with relatively heavy physical exertion, which increases oxygen consumption and exacerbates hypoxia.^28^^,^^29^ Construction tasks are also more likely to involve contact with dust, waste gas, and cold weather, thereby potentially increasing CMS risk. Furthermore, a previous study suggested that high-altitude residents were at higher risk for developing CMS in urbanized and industrialized regions.^15^ Our findings showed a similar tendency; urban communities had higher CMS rates than rural communities. However, this association is complex and likely involves social factors. Further sociomedical research is necessary to elucidate the effects of urbanization on CMS risk factors.

The availability of more primary care medical personnel, water purification systems, and timely hospital access did not significantly affect CMS rates in these immigrant communities. Smoking and drinking behaviors also had no significant effect on CMS rates, although smoking^30^ is considered to be a risk factor for CMS. We provide some possible reasons for these findings below.

Firstly, community smoking and drinking rates do not reflect the amounts of smoking and drinking, which thus introduces an ecological bias. Alternatively, the similarity of the rates in most communities in our study limited variation in CMS rates. Secondly, because CMS is a chronic, progressive, non-fatal disease and few CMS patients were hospitalized, hospital transfer time likely had little impact on CMS rates, despite the great variability among communities in hospital accessibility. Aside from a descent in altitude, the main treatment for CMS is drug therapy. If the medicine supply in a community is sufficient, patients need not go to a hospital. Thirdly, the insignificant effect of primary care medical personnel may have been due to the relatively equal distribution of healthcare human resources within the sample. Alternatively, this variable may be characterized by a threshold effect rather than a linear correlation, eg, the difference in the effect of 1 versus 2 primary care physicians might be negligible in a small community, whereas the presence or absence of primary care personnel might result in a noticeable difference.

### Limitations

The results of this study should be interpreted with caution for several reasons. Firstly, the use of results from ecological analysis to make inferences about residents of an area is subject to the ecological fallacy effect.^31^^,^^32^ For example, the quantity of oxygen intake from supply systems could not be measured in this study but may have influenced the association of oxygen intake with CMS rates. It was also difficult to incorporate community-level smoking and drinking quantities into our analyses, and some ecological biases in these variables are inevitable. Secondly, we collected data from 2 surveys conducted in different regions by different observers; although the diagnostic criteria and survey methods were the same, this factor may have introduced some bias. Thirdly, the data obtained using questionnaires and interviews may have been affected by recollection bias, and data extracted from military records were subject to some delay in information reporting. For example, the demographic characteristics may have been out of date. Moreover, this study focused on a specialized population of similarly aged men with comparable diets and behavior, which limits the generalizability of our results to other populations. Thus, a robust causal association between community-level factors and CMS rates could not be determined using an ecological study.

Furthermore, factors such as utilization of health services, care-seeking behaviors, and self-protection may also have affected CMS prevalence and were not fully considered in this study. These factors occur within institutional structures and can be estimated by examining socioeconomic status, features of the healthcare system, healthcare accessibility, education (general educational and special health instruction), and cultural beliefs.^33^^,^^34^

Although all Chinese servicemen enjoy access to free healthcare services, inequalities may exist between higher- and lower-ranking units, which could cause variation in utilization of health services. The use of medicine expenditure to represent healthcare utilization in this study may thus contain inherent biases. Cultural beliefs and educational status affect the recognition of illness severity and healthcare-seeking behavior. Because all military units in this study were assumed to share a similar cultural and educational background, we did not consider these factors. Nevertheless, further comparative studies of different civilian and military communities are needed. Healthcare systems in highland areas are frequently characterized by unequal distribution, limited medical resources, transportation difficulties, and isolation within small regions. These problems are exacerbated in remote frontier communities. In such contexts, healthcare delivery and accessibility are thus more complex factors than in settings with ready hospital access.

Self-protection is another important factor affecting CMS prevalence that may also be related to cultural beliefs and educational status. Cultural beliefs often lead to improper self-care and consultation with other laymen, which can result in delayed treatment seeking. In contrast, education has a positive impact on self-protection awareness and skills, such as respiratory capacity exercise, psychological self-regulation, control of smoking and drinking, reasonable oxygen utilization, and regular vitamin intake. This study treated these factors as similar in all communities in the sample, which might be imprecise.

Despite these limitations, our study serves as a useful complement to preliminary epidemiologic studies. By demonstrating associations between community-level factors and CMS rates, this study is a necessary first step in the identification of plausible contributing factors and improvement of health services in highland immigrant communities. This study should be considered as hypothesis-generating rather than definitive. To incorporate changes in health services over time, future studies should investigate these associations using longitudinal data.

## Conclusion

Although the principal risk factor for CMS is chronic exposure to high altitude, our study found associations between several community-level factors and CMS rates in a population of young male immigrants. To alleviate the burden of CMS among highland immigrants, policy-makers should prioritize investment in medicine supplies and the installation and maintenance of oxygen-supply equipment. Preventive interventions for construction workers should also be implemented. The effects of urbanization on CMS development should be examined in greater detail to detect sociomedical factors related to the prevalence of CMS.
